# Comparing implementation strategies for training and supervising nonspecialists in Group Problem Management Plus: A hybrid effectiveness-implementation trial in Colombia

**DOI:** 10.1017/gmh.2024.95

**Published:** 2024-10-22

**Authors:** M. Claire Greene, Diany Castellar, Manaswi Sangraula, Natalia Camargo, Jennifer Diaz, Valeria Meriño, Lucy Miller-Suchet, Ana Maria Chamorro Coneo, Marcela Venegas, Maria Cristobal, David Chávez, Brandon Kohrt, Peter Ventevogel, Miguel Uribe, Marilyn DeLuca, James Shultz, Zelde Espinel, Leslie Snider, Lisa Marsch, Sara Romero, Monica Ferrer, Abel Guerrero Gonzalez, Camilo Ramirez, Ana Maria Trejos Herrera, Matthew Schojan, Annie G. Bonz, Adam Brown

**Affiliations:** 1 Program on Forced Migration and Health, Heilbrunn Department of Population and Family Health, Columbia University Mailman School of Public Health, New York, NY, USA; 2 HIAS Colombia, Barranquilla, Colombia; 3 Trauma and Global Mental Health Lab, The New School of Social Research, New York, NY, USA; 4 Department of Psychology, Universidad del Norte, Barranquilla, Colombia; 5 HIAS Colombia, Bogotá, Colombia; 6 HIAS, Silver Spring, MD, USA; 7 HIAS Colombia, Cali, Colombia; 8 Psychiatry and Behavioral Health, George Washington School of Medicine and Health Sciences, Washington, DC, USA; 9 Public Health Section, United Nations High Commissioner for Refugees, Geneva, Switzerland; 10 Department of Psychiatry, Pontificia Universidad Javeriana, Bogotá, Colombia; 11 Department of Psychiatry, School of Medicine, New York University, New York, NY, USA; 12 Public Health Sciences, University of Miami, Miami, FL, USA; 13 Independent Consultant, Peace in Practice, Amsterdam, Netherlands; 14 Department of Psychiatry, Dartmouth College, Hanover, NH, USA; 15 Department of Global Health and Social Medicine, Harvard Medical School, Boston, MA, USA; 16 Department of Neurology, NYU Grossman School of Medicine, New York, NY, USA; 17 Department of Psychology, Universidad del Rosario, Bogotá, Colombia

**Keywords:** task sharing, Group PM+, migrants, refugees, Colombia

## Abstract

Migrants and refugees face elevated risks for mental health problems but have limited access to services. This study compared two strategies for training and supervising nonspecialists to deliver a scalable psychological intervention, Group Problem Management Plus (gPM+), in northern Colombia. Adult women who reported elevated psychological distress and functional impairment were randomized to receive gPM+ delivered by nonspecialists who received training and supervision by: 1) a psychologist (*specialized technical support*); or 2) a nonspecialist who had been trained as a trainer/supervisor (*nonspecialized technical support*). We examined effectiveness and implementation outcomes using a mixed-methods approach. Thirteen nonspecialists were trained as gPM+ facilitators and three were trained-as-trainers. We enrolled 128 women to participate in gPM+ across the two conditions. Intervention attendance was higher in the specialized technical support condition. The nonspecialized technical support condition demonstrated higher fidelity to gPM+ and lower cost of implementation. Other indicators of effectiveness, adoption and implementation were comparable between the two implementation strategies. These results suggest it is feasible to implement mental health interventions, like gPM+, using lower-resource, community-embedded task sharing models, while maintaining safety and fidelity. Further evidence from fully powered trials is needed to make definitive conclusions about the relative cost of these implementation strategies.

## Impact statement

Evidence supporting the effectiveness of mental health interventions delivered through task sharing is growing, yet most of this evidence is generated from efficacy and effectiveness trials that may lack the external validity to reflect the reality of routine service delivery. There is limited empirical evidence comparing strategies for promoting the implementation and adoption of these interventions. This study compares implementation strategies for training and supervision of nonspecialists in a mental health and psychosocial support intervention for refugees, migrants and vulnerable host communities. Migrant, refugee and host community women residing in communities in Barranquilla, Colombia, who reported elevated psychological distress and functional impairment were randomized to receive a group-based psychological intervention, Group Problem Management Plus (gPM+), delivered by nonspecialist providers who were trained and supervised under different conditions. The first condition involved direct training and supervision by specialists (i.e., psychologists). The second condition involved training and supervision by nonspecialist community members with experience as gPM+ providers who received training to become trainers and supervisors. Indicators of intervention effectiveness and most indicators of adoption and implementation were comparable across these two implementation strategies. The cost of implementation was lower and fidelity was higher in the condition where facilitators were trained and supervised by nonspecialists. This study outlines a lower-resource model for implementing a group-based psychological intervention through a community-based, task-sharing model and provides evidence supporting the sustained quality and fidelity of mental health and psychosocial supports interventions delivered through task sharing.

## Introduction

The number of displaced persons in Colombia is among the highest in the world. With approximately 3 million refugees and asylum seekers and more than 5 million internally displaced persons, over 10% of people residing in Colombia have been displaced due to conflict, disaster or economic and political crises (IDMC, 2023; UNHCR, 2023a). Such populations face increased risks for mental health problems, yet have limited access to services, which is partially due to the lack of mental health providers and the barriers refugees and migrants often face when seeking health services (Silove et al., 2017; Morina et al., 2018; Blackmore et al., 2020; Zambrano-Barragan et al., 2021; Patane et al., 2022; World Health Organization, 2022). Task sharing, the training of nonspecialists to provide mental health and psychosocial support (MHPSS), is an implementation strategy used to increase access to MHPSS (Cohen and Yaeger, 2021). Evidence from evaluations, often randomized controlled trials, have demonstrated that task sharing of MHPSS interventions can effectively address common mental health problems in displaced populations (van Ginneken et al., 2013; Barbui et al., 2020; Naslund and Karyotaki, 2021). However, there are challenges to task sharing that may preclude the sustainability of these models including intensive ongoing training and supervision, provider turnover, resources required to adopt interventions into routine practice and dynamic implementation contexts (Murray et al., 2014; Sijbrandij et al., 2017).

Problem Management Plus (PM+) is a scalable psychological intervention developed by the World Health Organization (WHO) designed to be delivered in a group or individual format by trained nonspecialists using a task-sharing model. PM+ is composed of evidence-based strategies including stress management, problem solving, behavioral activation and mobilizing social support (Acarturk et al., 2022). The group format, Group PM+ (gPM+), has demonstrated feasibility and/or effectiveness in reducing symptoms of common mental health problems in humanitarian settings and among migrant and refugee populations (Rahman et al., 2019; Sangraula et al., 2020; Jordans et al., 2021; Acarturk et al., 2022; Bryant et al., 2022a; Spaaij et al., 2022; de Graaff et al., 2023). Little is known about how these intervention effects translate into “real-world” implementation (Sijbrandij et al., 2017; Fuhr et al., 2020a,b).

Randomized trials are often accompanied by resources to support specialized, ongoing training and supervision, intensive quality monitoring and levels of technical support that may not reflect the resources available in real-world implementation. For example, high levels of training, supervision and support provided by specialist mental health providers is the norm in most trials and a barrier to the sustainability of task-sharing models (Murray et al., 2014). The apprenticeship model, a layered approach to supervision that builds the capacity of local supervisors who may not have specialized training to supervise local providers, has been developed to overcome the limitations of resource-intensive direct and ongoing supervision by specialists and reflect the resources more likely to be available in routine systems of care (Murray et al., 2011). This model may overcome other barriers, such as cultural and linguistic factors, that further reduce help-seeking and access to MHPSS. One concern is that this reduction in resources and intensive technical oversight will translate into a reduction in effectiveness when an intervention is transitioned into routine systems of care and implemented at scale (i.e., ‘voltage drop’; Bauer et al., 2015; Chambers et al., 2013). Empirically testing whether this voltage drop occurs by comparing outcomes of scalable interventions when delivered through “randomized controlled trial conditions” with supervision and technical support from specialists to “routine service delivery conditions” with technical support from nonspecialists is essential to bridging the research-to-practice gap.

The objective of this study was to examine the effectiveness and implementation of gPM+ when accompanied by intensive, specialized technical support compared to training, supervision and technical support provided by trained nonspecialists. Participants were women from communities that host many refugees and migrants in Barranquilla and Soledad, Colombia. We compared two implementation strategies (specialized vs. nonspecialized technical support) across the following outcomes: reach, effectiveness, adoption, implementation and maintenance (Glasgow et al., 1999; Holtrop et al., 2021).

## Methods

### Setting

Over 7 million people have fled the economic and political crisis in Venezuela to nearby Latin American countries (UNHCR, 2023a). Around 3 million Venezuelans currently live in Colombia, 51% of whom are women (GIFMM Colombia, 2023). More than 850,000 Colombians have returned from Venezuela since 2015 (UNHCR, 2023b). HIAS, an international NGO that supports refugees globally, has been working since 2019 to bridge the gap in community-based mental health needs of refugees, migrants and vulnerable host community members in Colombia.

This research was executed within three communities in Barranquilla and the municipality of Soledad, which are located on the Caribbean coast and the fourth largest settlement of Venezuelans in Colombia. The study communities included: 1) Primero de Mayo: a neighborhood in Soledad with high levels of violence, insecurity and extremely limited access to services; 2) Santa Maria: a neighborhood in Barranquilla hosting many Venezuelan refugees and migrants; and 3) Villa Caracas: an informal settlement with most residents being Venezuelan without regularized migration status.

### Participants and procedures

**Figure 1.**
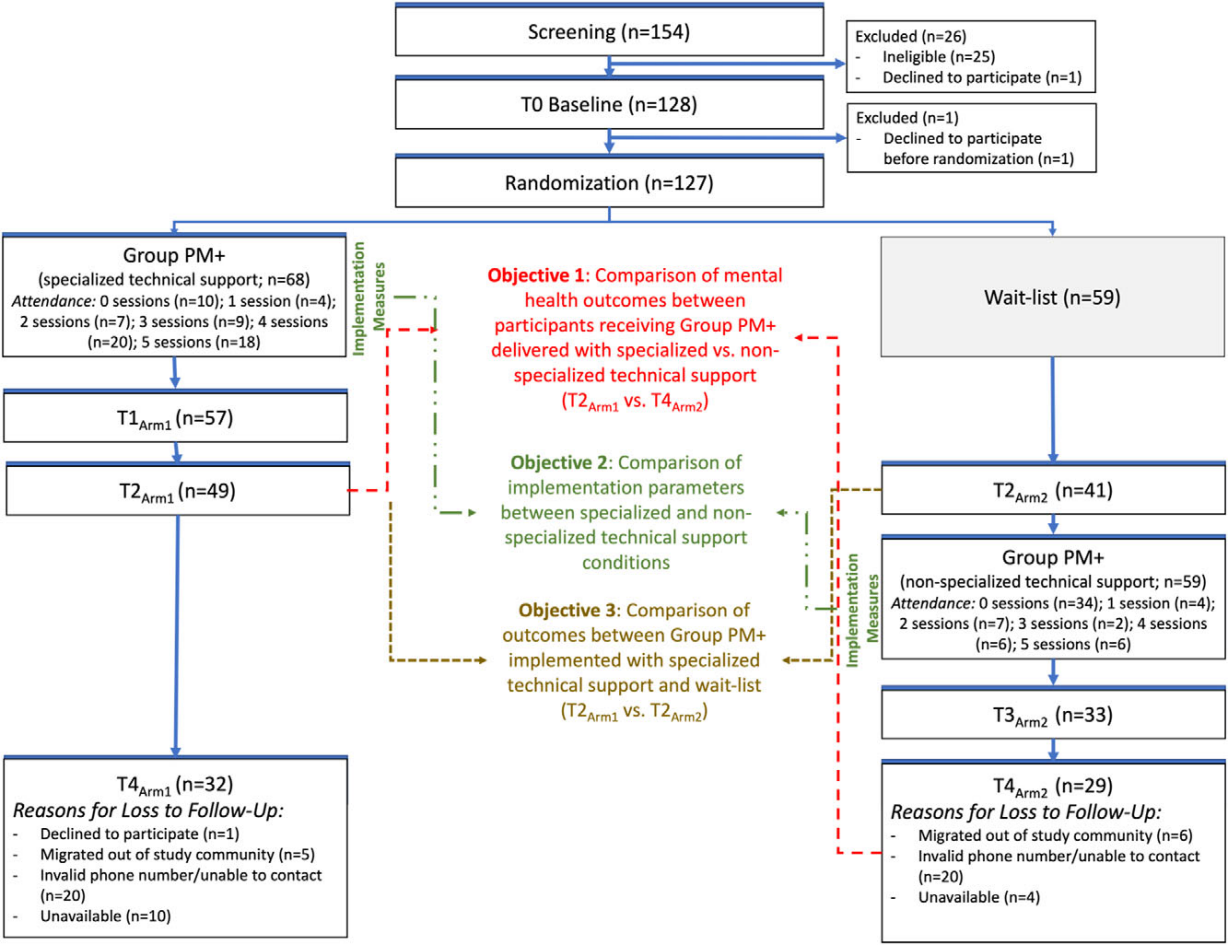
Participant flow diagram.

We conducted a hybrid effectiveness-implementation trial comparing gPM+ delivered through two task-sharing models with varying levels of technical support, training and supervision. All gPM+ participants were individually randomized using a random number generator to receive gPM+ within one of the study conditions and completed baseline, endline and follow-up assessments. While all participants were enrolled contemporaneously, the two study conditions were implemented sequentially similar to a crossover design whereby one condition received gPM+ immediately after enrollment and the comparison condition received gPM+ following a wait-list period (Figure 1) (Hemming et al., 2020). The study employed a non-inferiority evaluation framework to examine whether gPM+ delivered through task sharing with nonspecialized technical support was comparable in effectiveness and implementation to gPM+ delivered through task sharing with specialized technical support. All activities were conducted in Spanish. Additional details about the study design and procedures are described in the protocol (Sangraula et al., 2023).

#### Group Problem Management Plus intervention and implementation strategies

Group PM+ is a five-session intervention developed by the WHO to alleviate common mental health problems among adults exposed to adversity (Dawson et al., 2015). We adapted gPM+ through a review of the Spanish manual by local mental health professionals, humanitarian practitioners and community council members (Sangraula et al., 2021). Pre-implementation adaptations included modifying elements of gPM+ implementation to fit the study context, adjusting language and images used in the intervention materials, specifying safe and appropriate community engagement strategies and selecting and matching facilitators to participant preferences (Supplementary Table 3 in the Supplementary Material).

Adapted gPM+ was delivered to all participants using one of two implementation strategies (Table 1): 1) *task sharing with specialized technical support*, involving gPM+ delivered by nonspecialists facilitators from the study communities who were trained and supervised by a clinical psychologist; and 2) *task sharing with nonspecialized technical support*, involving gPM+ delivered by nonspecialist facilitators from the study communities who were trained and supervised by nonspecialists who had completed a training of trainers. Nonspecialist trainers were selected by clinical supervisors from among the cadre of previous gPM+ facilitators based on their interest in becoming a trainer, competency levels and fidelity to gPM+. Nonspecialists who were trained-as-trainers received training and biweekly supervision from a clinical psychologist to monitor safety. Trainings lasted 8 days for facilitators and 5 days for trainers. Trainings for facilitators and trainers included didactic components, roleplays and observations. All facilitators conducted practice groups prior to implementation of gPM+ for the trial.

**Table 1.** Implementation strategy specification

	Task sharing with specialized technical support	Task sharing with nonspecialized technical support
Actor	*Trainer/supervisor:* Clinical psychologist (study investigator) provides training and supervision. All sessions supervised by psychologists from implementing organization. *Group PM+ facilitator:* Nonspecialists from study communities	*Trainer/supervisor:* Nonspecialists from study communities who had been Group PM+ facilitators and were trained as trainers. All sessions were supervised by nonspecialist trainers. Staff from implementing organization also attended sessions to ensure safety and overall fidelity of the intervention. *Group PM+ facilitator:* Nonspecialists from study communities.
Action	Women from the study communities are selected by the implementing organization to participate in a training delivered by a clinical psychologist to become Group PM+ facilitators. After completing the training, facilitators deliver the intervention to groups of approximately eight eligible women with moderate functional impairment and psychological distress. Group PM+ facilitators receive weekly supervision from the clinical psychologist and all sessions were supervised and supported by psychologists from the implementing organization.	Women who had previously been trained as Group PM+ facilitators and delivered the intervention in their communities are trained by the implementing organization (including psychologists) to become Group PM+ trainers/supervisors (i.e., a training–of–trainers). These new trainers/supervisors select migrant or refugee women from their community who they train to become Group PM+ facilitators. After completing the training, the newly trained facilitators deliver the intervention to groups of approximately eight eligible women with moderate functional impairment and psychological distress. Group PM+ facilitators receive weekly supervision from the new trainers/supervisors (nonspecialists). All sessions were supervised and supported by the new trainers/supervisors, who received biweekly supervision from the clinical psychologist to monitor safety.
Target	*Group PM+ facilitators:* Venezuelan women from the study community who are recognized by the implementing organization as active members or leaders in their community *Group PM+ participants*: Women with moderate psychological distress and functional impairment	*Group PM+ facilitators:* Venezuelan women from the study community who are identified by newly trained trainers/supervisors to have the necessary skills to become a facilitator *Group PM+ participants:* Women with moderate psychological distress and functional impairment
Temporality	*Training:* Training of facilitators; Once (pre–implementation) *Supervision:* Weekly (implementation) *Intervention implementation:* Weekly (implementation)	*Training:* Training–of–trainers followed by training of facilitators; Once (pre–implementation) *Supervision:* Weekly (implementation) *Intervention implementation:* Weekly (implementation)
Dose	*Training:* Training of facilitators: 2 weeks *Supervision:* 1–2 h per week outside of sessions; All sessions were supervised *Intervention implementation:* 1.5–2 h per session	*Training:* Training of trainers: 1 week; Training of facilitators: 2 weeks *Supervision:* 1–2 h per week outside of sessions; All sessions were supervised *Intervention implementation:* 1.5–2 h per session
Outcomes affected	Reach: Reach, retention, sample characteristics Effectiveness: Non–inferior effect sizes from baseline to follow–up in client mental health outcomes (PHQ–9, GAD–7, PCL–5, PSYCHLOPS, AUDIT) Adoption: Completion of training and facilitator competencies, adherence to supervision, intervention fidelity Implementation: Adaptations to the intervention and its implementation Maintenance: Cost, potential for scalability, sustainability; sustainability planning	
Justification	The objective of this comparison is to examine whether Group PM+ can be delivered as effectively without specialized, external technical support and the resources that are often available within controlled research studies. Changes in how facilitators are trained and supervised contributes to “the voltage drop” in effectiveness in real world settings. Comparing Group PM+ implemented with specialized vs. nonspecialized training, supervision and technical support can inform the implications of translating the evidence on the effectiveness and implementation of Group PM+ from research to practice.	

#### Enrollment and assessments

Group PM+ participants included women 18+ years of age, living in one of three communities in Barranquilla and Soledad, reporting an intention to stay in the study community for at least 3 months, and reporting moderate functional impairment (WHO Disability Assessment Schedule 2.0, WHODAS>16; Üstün et al., 2010) and moderate psychological distress (General Health Questionnaire, GHQ-12>2; Goldberg, 1988). We did not restrict the sample based on migration status or nationality and thereby included refugees, migrants, asylum seekers, internally displaced persons, returnees and host community members. Enrolled participants who provided written informed consent were invited to complete a baseline interview and were randomized to one of the study conditions. All participants completed follow-up assessments at endline (i.e., 1 week after gPM+), 3-months and 6-months post-enrollment. Participants allocated to the specialized technical support condition were assigned to a gPM+ group immediately following the baseline assessment. Participants allocated to the nonspecialized technical support condition were assigned to a gPM+ group following their 3-month post-enrollment assessment. At the end of the study, we conducted one focus group in each study community with gPM+ participants. Group PM+ facilitators and supervisors also participated in qualitative interviews post-training and focus groups post-implementation to explore implementation barriers, facilitators and outcomes.

The study achieved its target sample size of 128 participants or approximately eight groups of eight participants per study condition. This sample size enabled us to determine non-inferiority between the two study conditions with a large margin, but is underpowered to detect small to moderate differences.

#### Measures

The primary client-level outcome was depressive symptoms, which was measured using the Patient Health Questionnaire (PHQ-9; Spitzer et al., 1999) among all participants at all timepoints (baseline, endline, 3-month and 6-month post-enrollment). The PHQ-9 has been translated into Spanish and validated in Colombia (Cassiani-Miranda et al., 2021). Secondary outcomes were assessed at baseline, endline and 3-month follow-up for all participants as well as at the 6-month follow-up in the nonspecialized technical support condition: post-traumatic stress symptoms (PTSD Checklist for DSM-5, PCL-5; Ashworth et al., 2006; Weathers et al., 2013), self-identified problems (Psychological Outcomes Profile, PSYCHLOPS), harmful alcohol use (Alcohol Use Disorders Identification Test, AUDIT; Saunders et al., 1993), migration-related distress (Post-Migration Living Difficulties, PMLD; Tay et al., 2019) and COVID-19 related stress (Perceived Stress Scale, PSS-10). Migration-related distress was not measured among participants from the host community. Additional details can be found in Supplementary Table 5 in the Supplementary Material.

Service-level outcomes included attendance and completion of intervention sessions, training and supervision by supervisors and facilitators; facilitator competencies during training and implementation; and safety concerns or adverse events. Competencies were assessed using the Enhancing Assessment of Common Therapeutic factors (ENACT) rating scale. The ENACT Basic Helping Skills assessment was collected pre-training (Kohrt et al., 2015). The ENACT for PM+ competencies was collected post-training (Pedersen et al., 2021a). The GroupACT, which evaluates group facilitation competencies, was collected through observation during sessions by supervisors (Pedersen et al., 2021b).

Implementation-level outcomes included reach, retention, fidelity, acceptability, feasibility and cost of implementation. Reach and retention were measured through routine monitoring forms and explored through qualitative interviews. We explored the acceptability and feasibility of gPM+ through adaptations documented using the *Bernal Framework* and exploring these outcomes through qualitative interviews and focus groups (Bernal et al., 2009). Cost was estimated using program expenses. Fidelity to the intervention was assessed by a clinical supervisor or lay trainer using a gPM+ checklist completed during each session (Sangraula et al., 2020). A summary of implementation outcomes is provided in Table 2.

**Table 2.** Implementation outcomes

RE-AIM domain Research question	Indicator(s)	Study findings			
		Overall	Specialized training/supervision condition	Nonspecialized training/supervision condition	Test statistic (*p*-value)
Reach Can Group PM+ reach and retain as many participants in the intervention when delivered with specialized vs. nonspecialized technical support, training and supervision?	Rate of recruitment Participants recruited per week	5.40	–	–	–
	Intervention engagement Participants who attended at least one session	83 (65.35)	58 (85.29)	25 (42.37)	25.70 (<.001)
	Retention				
	Intervention completion (i.e., completed more than three sessions)	61 (48.03)	47 (69.12)	14 (23.73)	26.07 (<.001)
	Intervention completion among those who attended at least one session	61 (73.49)	47 (81.03)	14 (56.00)	5.62 (0.018)
	Mean pre–intervention depressive symptoms	7.46 (5.33)	8.03 (5.26)	6.51 (5.37)	−1.45 (0.151)
Effectiveness Does Group PM+ yield comparable changes in psychological distress when delivered with specialized vs. nonspecialized technical support, training and supervision?	Non–inferior effect sizes Difference in change from pre–intervention to endline and 3–month follow–up in primary (PHQ–9) and secondary (PCL–5, PSYCHLOPS, AUDIT, PMLDC, PSS) outcomes	See Supplementary Table 2 in the Supplementary Material Significant within–group reductions in depressive symptoms, PTSD symptoms and migration–related distress from baseline to endline in both study conditions. Significant within–group reductions in personally identified problems from baseline to endline in specialized technical support condition only. No significant between–group differences from baseline to 3–months post–intervention (calendar or exposure time).			
Adoption Do lay facilitators and trainers continue to apply Group PM+ as intended when delivered with specialized vs. standard levels of technical support, training and supervision?	Facilitator engagement and retention				
	Facilitators selected for training	20	10	10	–
	Facilitators who began training	16 (80.0%)	8 (80.0%)	8 (80.0%)	–
	Facilitators who completed training (among those who started)	13 (81.3%)	7 (87.5%)	6 (75.0%)	–
	Facilitators who attended all supervision sessions	13 (100.0%)	7 (100.0%)	6 (100.0%)	–
	Facilitators who attended all intervention sessions	13 (100.0%)	7 (100.0%)	6 (100.0%)	–
	Facilitator competency pre– and post–training^a^ Pre–training ENACT Basic Helping Skills scores	1.99 (0.24)	1.95 (0.35)	2.01 (0.15)	−0.31 (0.760)
	Pre–training ENACT Average Number of Level 1	2.54 (2.11)	1.83 (1.84) range: 0–5	1.43 (1.13) Range: 0–3	0.93 (0.392)
	Post–training ENACT PM+ Competency scores	2.14 (0.13)	2.06 (0.10)	2.18 (0.15)	−1.70 (0.119)
	Competency across session implementation				
	GroupACT (*n* = 44 sessions in specialized condition, *n* = 39 sessions in nonspecialized condition)	2.61 (0.63)	2.79 (0.68)	2.40 (0.50)	1.22 (0.216)
	GroupACT session 1	2.41 (0.54)	2.44 (0.68)	2.38 (0.37)	0.24 (0.812)
	GroupACT session 2	2.54 (0.55)	2.76 (0.58)	2.29 (0.43)	1.90 (0.073)
	GroupACT session 3	2.73 (0.66)	2.94 (0.74)	2.50 (0.50)	1.43 (0.173)
	GroupACT session 4	2.55 (0.73)	2.87 (0.71)	2.37 (0.69)	1.43 (0.177)
	GroupACT session 5	2.72 (0.68)	3.00 (0.72)	2.44 (0.55)	1.72 (0.108)
	Fidelity to intervention implementation				
	Mean fidelity score (overall)	0.70 (0.15)	0.64 (0.15)	0.78 (0.10)	−2.29 (0.037)
	Mean fidelity score (session 1)	0.75 (0.21)	0.67 (0.24)	0.84 (0.15)	−1.75 (0.101)
	Mean fidelity score (session 2)	0.67 (0.15)	0.59 (0.14)	0.76 (0.09)	−2.91 (0.011)
	Mean fidelity score (session 3)	0.69 (0.20)	0.61 (0.21)	0.77 (0.15)	−1.82 (0.090)
	Mean fidelity score (session 4)	0.69 (0.18)	0.62 (0.17)	0.78 (0.16)	−1.97 (0.069)
	Mean fidelity score (session 5)	0.71 (0.15)	0.69 (0.16)	0.74 (0.14)	−0.80 (0.438)
Implementation Does the acceptability and feasibility of Group PM+ differ when delivered with specialized vs. nonspecialized technical support, training and supervision?	Adaptations to implementation	See Supplementary Table 3 in the Supplementary Material Most adaptations were made pre–implementation (87.5%); targeted characteristics of the intervention (25.0%) or facilitators (25.0%); and related to the context (25.0%), language (18.8%), or people involved in the intervention (18.8%).			
	Safety No. of adverse events or serious adverse events	0	0	0	–
Maintenance Is Group PM+ sustainable in the absence of specialized technical support, training and supervision?	Cost of PM+ implementation (in USD) Total cost of implementation	67,393 Total study cost	40,785 Cost per arm	26,608 Cost per arm	–
	Training costs	5,803	1,900	3,903	–
	Program personnel costs (trainers, supervisors, facilitators, project coordinators)	25,542	21,190	4,352	–
	Research personnel costs (research assistants)	31,728	16,800	14,928	–
	Assessments	590	295	295	–
	Materials and supplies	3,730	600	3,130	–

a Pre-training ENACT scores were collected for six trainees in the specialized training/supervision condition and for seven trainees in the nonspecialized condition. ENACT score for one trainee in the specialized training/supervision condition was not able to be analyzed because of technical issues with the video recording during the pre-training assessment. Post-training ENACT PM+ competency scores were collected from three trainees in the specialized training/supervision condition and for all seven trainees in the nonspecialized condition.

### Analysis

Quantitative analyses of client-level outcomes were conducted using mixed effects models to account for the clustering of observations within participants and communities. To examine the comparative effectiveness of gPM+ delivered using two different implementation strategies, we estimated the interaction between study condition and time from pre-implementation (T0_Arm1_, T2_Arm2_) to endline (T1_Arm1_, T3_Arm2_) and 3-months post-implementation (T2_Arm1_, T4_Arm2_). To examine the effectiveness of gPM+ relative to waitlist, we estimated the interaction between study condition and time from baseline (T0) to 3-months post-implementation (T2). We estimated the unadjusted difference in change over time in participant outcomes between study conditions and constructed multivariable models adjusting for age and predictors of attrition: employment and socioeconomic indicators, time in community, household size and history of depression or anxiety. We conducted three sensitivity analyses to evaluate the robustness of results: 1) per protocol analysis of intervention completers (i.e., attended more than three sessions); 2) analysis among participants with elevated mental health symptoms prior to implementation (i.e., did not remit during waitlist period; PHQ-9≥5 or PCL-5≥30); 3) matched comparison between study conditions at the pre-implementation assessment using propensity score matching (T0_Arm1_, T2_Arm2_). Quantitative service- and implementation-level outcomes were compared between study conditions using chi-squared and *t*-tests. All quantitative analyses were conducted in Stata Version 18.

Qualitative data were analyzed using thematic analysis (Braun and Clarke, 2014). All interviews were coded by two independent researchers in Dedoose. Discrepancies were resolved by team discussion. After coding, the analysis team prepared memos summarizing study codes and themes. We organized and integrated the quantitative and qualitative findings according to the RE-AIM framework (Glasgow et al., 1999).

### Ethics and trial registration

All participants provided written informed consent prior to enrollment. Study procedures were approved by the IRB and Universidad del Norte in Barranquilla (#237) and Columbia University in New York (AAAU3933). The study was retrospectively registered on clinicaltrials.gov (https://clinicaltrials.gov/study/NCT05477355; Sangraula et al., 2023).

## Results

### Reach

Approximately 5.4 participants were recruited per week between January 31 and July 15, 2022, to achieve the target sample size of 128 participants. Most participants who were screened (*n* = 154) were eligible (*n* = 129) and one eligible participant declined to participate prior to enrollment (*n* = 1). One participant withdrew after completing the baseline and prior to randomization, resulting in 127 participants randomized to the study conditions. Enrolled participants were 33.3 years of age (SD = 10.7), on average, and most had a primary school education or higher (92.2%) and were married or living with their partner (54.3%). At baseline, most participants had a source of income through informal labor (23.8%), salaried or formal work (7.9%) or self-employment (19.8%). Most participants were Venezuelan (83.2%). Most participants self-identified as a migrant, refugee, or asylum seeker (88.2%); of whom one-quarter reported having an irregular migration status (26.4%). Colombian participants identified as a member of the host community (6.3%) internally displaced (3.2%), or a returnee (2.4%). Most participants had not previously utilized MHPSS services (80.2%). We did not identify large differences between study conditions in demographic factors or depressive symptoms at baseline (Supplementary Table 1 in the Supplementary Material).

We observed differences across study conditions in intervention engagement. Participants allocated to the specialized technical support condition who were immediately assigned to a gPM+ session were more likely to attend at least one session (85.3%) than were participants allocated to the nonspecialized technical support intervention who began gPM+ approximately 3 months after their baseline assessment (42.4%; *p* < 0.001). Intervention retention differed across study conditions. The proportion of participants who completed gPM+ (i.e., attended more than three sessions) was higher in the specialized technical support condition (69.1%) relative to the nonspecialized technical support condition (23.7%; *p* < 0.001). Participants were more likely to complete gPM+ if they did not have a cement house (i.e., indicator of lower socioeconomic status, OR = 3.01, *p* = 0.003), were living with their partner (vs. single, OR = 4.39, *p* = 0.001), or were self-employed (vs. unemployed, OR = 4.12, *p* = 0.047).

Participants reported several barriers to engaging in gPM+ including illness, family responsibilities and childcare, difficulties managing other responsibilities and extreme weather conditions. Participants explained that their motivation to participate, having the sessions in a convenient location or providing transportation, and the ability to bring young children and/or find childcare were key facilitators to engaging in gPM+. Effective and consistent communication from facilitators and group members was a strategy that promoted retention. Most focus group participants who had not attended all sessions reported that they found gPM+ useful, but faced barriers to continued participation.

*“In the first session I learned a lot. I didn’t know the problems I faced existed. I was curious and liked [gPM+] which is why I kept coming back. Of course, I have my child, so sometimes it is difficult for me, but the responsibility comes first…’”*

- Participant, Santa Maria

### Effectiveness

We observed significant within-group reductions in depressive symptoms (specialized = −2.21, 95% CI: −3.97, −0.45; nonspecialized = −2.39, 95% CI: −4.63, −0.15), PTSD symptoms (specialized = −5.79, 95% CI: −10.54, −1.04; nonspecialized = −6.55, 95% CI: −11.85, −1.24), migration-related distress (specialized = −3.36, 95% CI: −6.71, −0.01; nonspecialized = −5.62, 95% CI: −9.07, −2.17) and COVID-19 related stress (specialized = −3.46, 95% CI: −5.88, −1.03; nonspecialized = −4.67, 95% CI: −7.46, −1.88) from baseline (T0) to endline (T1_Arm1_, T3_Arm2_) in both study conditions (Supplementary Table 2 in the Supplementary Material). Significant reductions in PTSD symptoms (−5.94, 95% CI: −11.60, −0.27), migration-related distress (−4.93, 95% CI: −8.86, −1.01) and COVID-19 related stress (−4.55, 95% CI: −7.19, −1.91) persisted at 3-months post-implementation in the specialized technical support condition only. Personally identified problems reduced from baseline to endline in the specialized technical support condition only (−1.80, 95% CI: −3.54, −0.05). Focus group participants described the changes they experienced through the program, which aligned with the purported gPM+ mechanisms of change, including their ability to manage problems through improved communication, patience, being proactive, stress management skills and social support.

*“I had fallen into a cycle of inactivity in which I did not go out, I would stop at home, it was just a fight with the children, with my partner. I did not get dressed up. I was a bit crazy all the time, stressed, fighting, I would say rude things and [gPM+] has helped me to control myself a little bit now. I mean, I get dressed up, I go out with my partner, I go out with the kids, we go to the park, we eat, and now I feel better, freer, more active, and calmer.”*

– Participant, Villa Caracas

**Figure 2.**
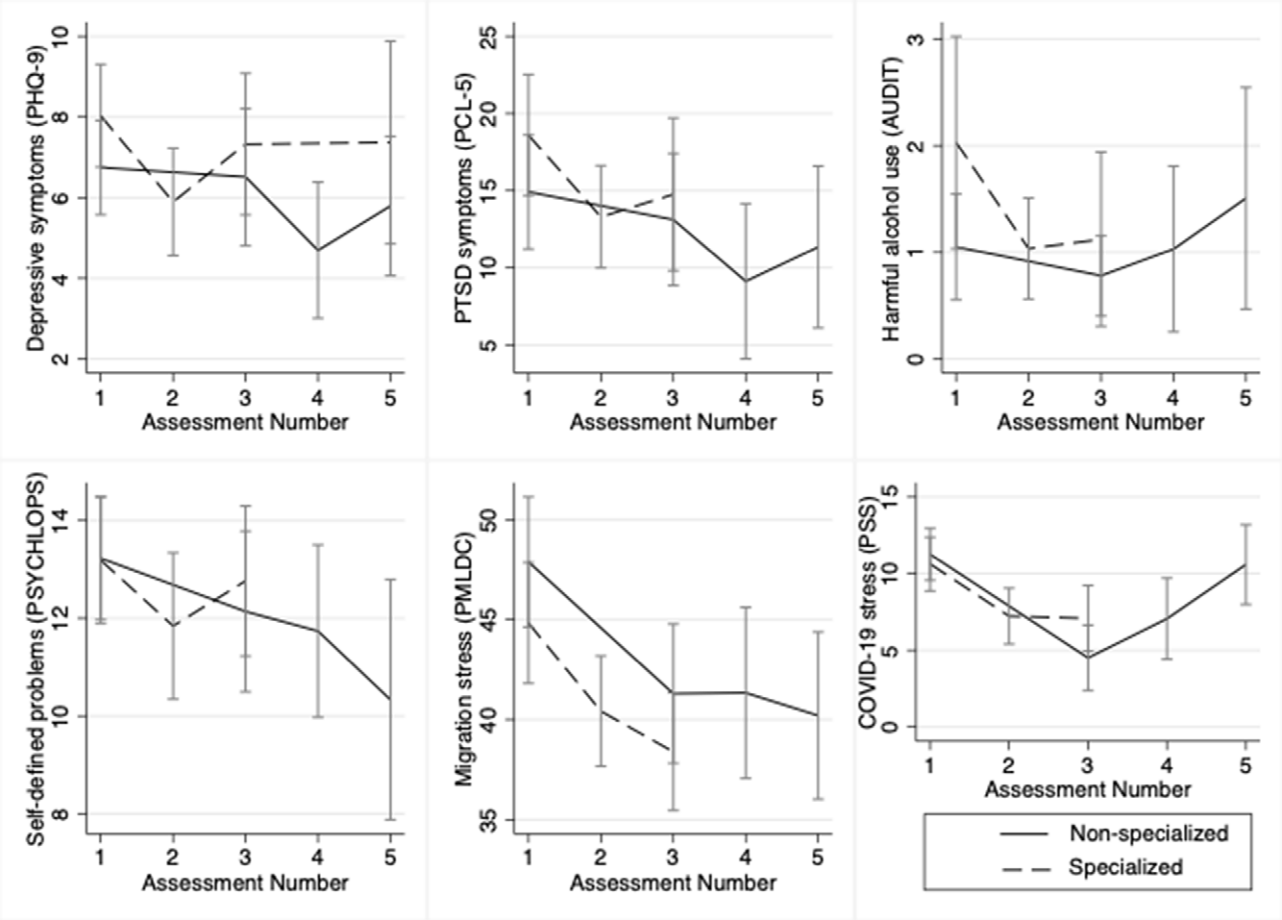
Change in participant outcomes over calendar time.

**Figure 3.**
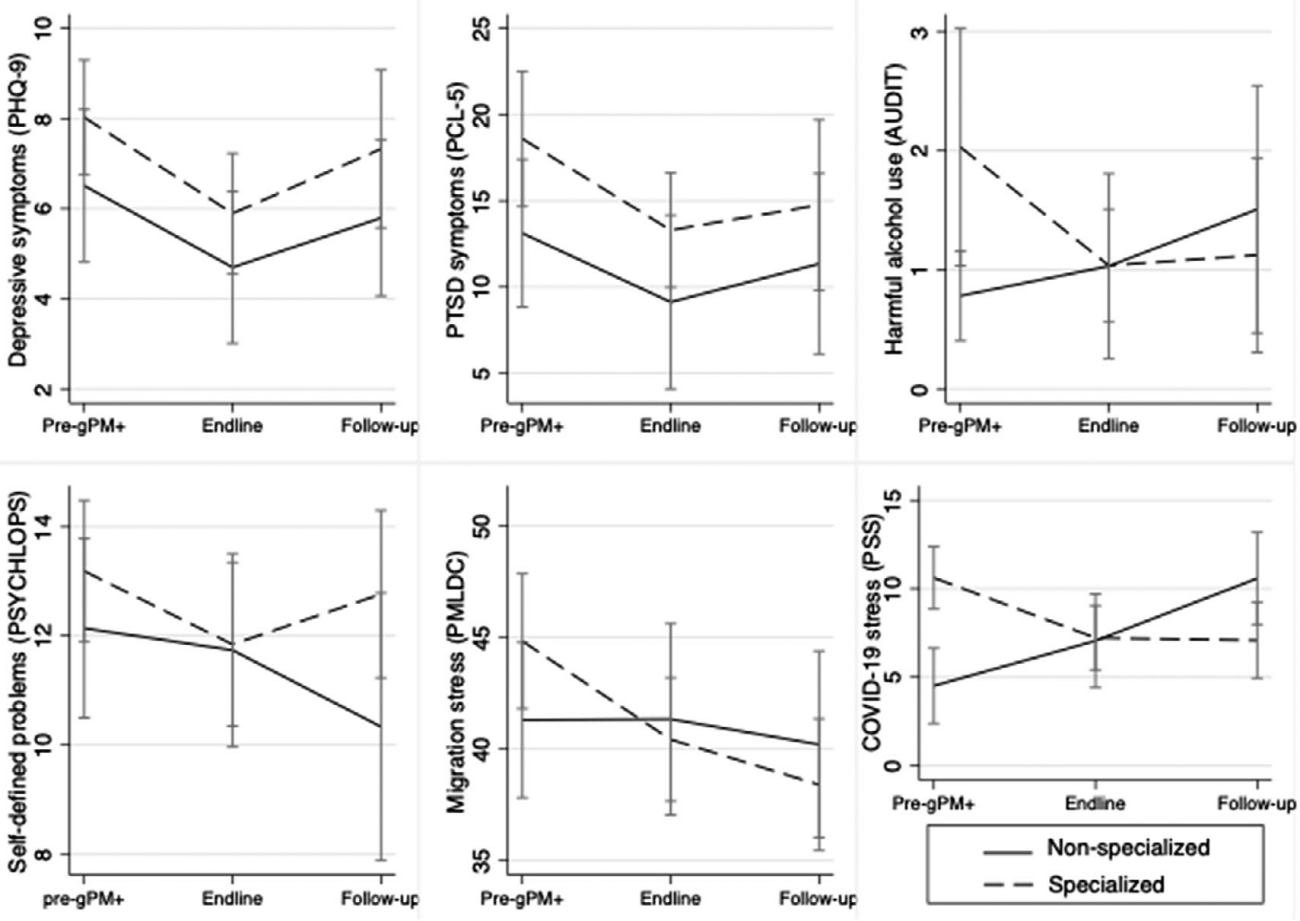
Change in participant outcomes over exposure time.

We did not observe significant differences from baseline to 3-months post-intervention between gPM+ (specialized) and wait-list control (Figure 2). Most endline and 3-month outcomes did not differ by the two implementation strategies (i.e., gPM+ delivered through specialized vs. nonspecialized technical support; Figure 3) except for COVID-19 stress and harmful alcohol use. We observed a greater reduction in COVID-19 stress at endline (−5.94, 95% CI: −9.57, −2.31) and 3 months (−5.03, 95% CI: −6.92, −3.14) relative to the nonspecialized condition. These results were similar in adjusted models, the per protocol analysis, and the sensitivity analysis restricted to participants who met cutoffs for depression or PTSD symptoms prior to gPM+. Propensity score models revealed significantly higher depressive (average treatment effect = 1.70, 95% CI: 0.15, 3.26) and PTSD symptoms (average treatment effect = 2.41, 95% CI: 1.62, 3.63) in the specialized technical support as compared to the nonspecialized technical support condition at endline, but this difference did not persist at the 3-month follow-up.

### Adoption

*Specialized technical support condition:* Ten Venezuelan women from the study communities who were recognized by HIAS as active members/leaders in their community were invited to participate in the gPM+ facilitator training provided by two psychologists. Of those invited, eight began the training and seven completed the training. Six of those who completed the training were Venezuelan and one was Colombian.

*Nonspecialized technical support:* Three of these Venezuelan facilitators were selected to be trained-as-trainers by clinical psychologists upon completion of gPM+ implementation. These three facilitators who became trainers, invited 10 nonspecialists from their community who they perceived to have the necessary skills to become a facilitator to participate in a second facilitator training that was led by these three newly trained trainers. Of those invited, eight began the training and six completed the training. Four of these facilitators were Venezuelan and the remaining two were Colombian-Venezuelan.

Competencies did not differ between study conditions for pre-implementation basic helping skills (specialized = 1.95, SD = 0.35; nonspecialized = 2.01, SD = 0.15), or post-training PM+ competencies (specialized = 2.06, SD = 0.10; nonspecialized = 2.18, SD = 0.15), or group facilitation competencies (specialized = 2.79, SD = 0.68; nonspecialized = 2.40, SD = 0.50). We observed higher fidelity to the intervention across sessions in the nonspecialized support condition (78% fidelity to session content) compared to the specialized support condition (64% fidelity; *p* = 0.037). We did not observe any serious adverse events during the study period in either condition.

In focus groups, facilitators shared the importance of practicing gPM+ strategies to fully comprehend and use them. Facilitators reported feeling nervous delivering gPM+, but their confidence improved through practice. Having prior experience as a participant in gPM+ or other MHPSS interventions made it easier to serve as facilitators. Feeling empowered, confident and motivated to help other women in their community ultimately encouraged them to become a facilitator.

*“I was motivated by the change in my life… When I started, I already had many problems and the program came to help me and motivated me to help other women in my community who were going through the same thing… If we can try to take those strategies to get out of [our problems] and to be better women for our children, for our partner and for our environment… That was one of the motivations, to stand up and tell them we can better women and we can face those problems that afflict us on a daily basis.”*

–Facilitator

### Implementation

Facilitators described several implementation challenges during focus groups including difficulty teaching gPM+ strategies to participants with different levels, participant hesitation to share personal experiences in a group setting due to confidentiality concerns, and difficulty implementing gPM+ alone (vs. in pairs as was done during training practice sessions). There were external factors (e.g., poor weather, competing priorities) that presented challenges and demotivated participants to attend sessions.

We documented multiple adaptations to gPM+ and its implementation throughout the study. Most adaptations were made pre-implementation (87.5%; Supplementary Table 3 in the Supplementary Material). The primary adaptation to the intervention made during implementation involved creating new intervention materials to promote visibility within the community and retention of study participants. In focus groups, participants acknowledged that materials were important, but ultimately secondary to the way facilitators and project staff treated the participant.

*“Our facilitator was very patient, very responsible… She was always there and affectionate, she supported us, she listened to us, we shared a lot with her, we laughed… She was always looking out for us in the group so that we would be there, so that we would participate, so that we would always be united.”*

–Participant, Primero de Mayo

### Maintenance

Facilitators and participants expressed a desire for gPM+ to continue to be offered within their communities after the close of the study.

*“We are not the only vulnerable people in the world. There are many people who need another person to listen to them and another person to talk to them, to support them, to understand them, to know that they are there at this moment for any situation in need of something in a moment of difficulty. And we are women, we understand what other women go through in their daily lives.”*

–Participant, Primero de Mayo

Facilitators described strategies that would promote program sustainability and enable them to provide gPM+ to more people within their community. Having proper certification and support from established organizations can assist with gaining trust and acceptance.

*“Many times without certification, without a license, people will say, “who are you?” Not everyone will open the door.”*

–Facilitator

Facilitators noted the need for financial resources to sustain gPM+. Implementation of the specialized technical support condition cost $40,785 USD in total as compared to $26,608 USD in the nonspecialized support condition. The primary difference in cost was driven by higher personnel costs in the specialized support condition ($21,190) compared to the nonspecialized support condition ($4,352). Other training costs were higher in the nonspecialized ($3,903) as compared to the specialized support condition ($1,900) because the new trainers had to secure additional materials and rent a space for the training. The cost of materials and supplies was higher in the nonspecialized ($3,130) as compared to the specialized support condition ($600) because the trainers and facilitators implementing gPM+ within the nonspecialized support condition generated new ideas for materials and resources to facilitate implementation (Supplementary Table 4 in the Supplementary Material).

Participants expressed the sustained impact of the program on their wellbeing and their desire to continue meeting with their groups and learn new information. They described how they have continued to apply the strategies within their lives, and they are better equipped to address future problems.

*“I wondered how [the strategies] would be part of our daily life, breathing, because all of the time we are stressed by work, by the children, by the house, by cleaning so we have to have the strategies with us all of the time. Breathing, for example, for stress.”*

–Participant, Primero de Mayo

## Discussion

This study evaluated the implementation of gPM+ delivered with training and supervision provided by a specialist as compared to a nonspecialist community member who had been trained as a gPM+ trainer/supervisor to determine whether gPM+ implementation could be maintained using routinely available, community-based resources. Across most indicators of effectiveness, adoption and implementation, these two conditions were largely equivalent. Participant retention and reductions in COVID-19 stress were higher in the specialized gPM+ condition, which may be confounded by the 3-month run-in period from enrollment for the nonspecialized support condition. The primary effectiveness analysis did not indicate significant differences between study conditions on changes in depressive symptoms. The nonspecialized support condition demonstrated higher levels of fidelity to the intervention and lower cost of implementation. Taken together, this study provides preliminary evidence that training nonspecialists as trainers and supervisors may enable cost-savings without compromising fidelity, safety, quality or effectiveness. However, further evidence from fully powered implementation trials is needed to make conclusions about the relative comparative and cost-effectiveness of these implementation strategies for gPM+ and similar interventions.

This study is among the first to test the implementation of gPM+ or similar MHPSS using a nonspecialist training and supervision model. Most previous studies of gPM+, including in Latin America, have utilized psychologists or other specialists as trainers and supervisors (Coleman et al., 2021). This study displays similarities to previous studies of gPM+. First, many of the adaptations made to the gPM+ manual were similar to previous PM+ studies, including adjusting language and images, modifying examples to reflect the type of adversity experienced by the target population, and presenting concepts related to mental health that are acceptable and consistent with how psychological distress is expressed within the study context (Perera et al., 2020; Coleman et al., 2021; Sangraula et al., 2021).

Second, many of the effectiveness and implementation outcomes were similar to previous gPM+ studies. Similar to previous studies, we found that it was feasible to train nonspecialists as gPM+ facilitators and they achieved sufficient competence through training (Khan et al., 2019; Sangraula et al., 2020), and we observed small to moderate reductions in depressive and PTSD symptoms during the intervention period that attenuated during the post-intervention period (Jordans et al., 2021; Bryant et al., 2022b). Other gPM+ trials have demonstrated sustained benefits of the intervention during the follow-up period (Rahman et al., 2019), suggesting that sustained effects are possible and future research is needed to understand who continues to benefit from gPM+ and how to promote these enduring impacts. Additionally, within-group intervention changes in mental health outcomes were small and most did not persist over time. The exceptions were PTSD, migration-related distress and COVID-19 stress, which remained significantly lower at the 3-month follow-up relative to baseline in the specialized condition. This reduction was not significantly larger than the reduction observed in the nonspecialized condition. However, our study was underpowered to detect small to moderate effects. Further research into such sustained effects on secondary outcomes is therefore warranted.

Third, many of the barriers to delivering and participating in gPM+ resembled previous studies of gPM+ and other community-based MHPSS interventions implemented in Latin America. Competing responsibilities, weather conditions, illness and inconvenient location and scheduling of the sessions were some of the common barriers reported by participants in this study (Khan et al., 2019; Shultz et al., 2019; Perera et al., 2022; Greene et al., 2023; Woodward et al., 2023). Having a member of the community serve as the facilitator and delivering the intervention within community settings mitigated some of these challenges as well as other barriers such as mistrust (Khan et al., 2019).

We also observed differences between the current study and previous studies of gPM+. Intervention attendance and study retention rates were higher in previous gPM+ trials, which may partially explain the smaller within-group effect sizes in this study compared to prior ones (Rahman et al., 2019; Jordans et al., 2021; Acarturk et al., 2022; Bryant et al., 2022a). The study’s context, concurrent COVID-19 pandemic, population and research design may have also contributed to these differences. Migrants and refugees are highly mobile populations and many participants relocated outside the study’s area or became unreachable during the implementation period. Attendance and study retention rates are more comparable to previous studies of other psychosocial interventions that have been conducted among displaced and migrant communities in Colombia (Bonilla-Escobar et al., 2018; Shultz et al., 2019) and nearby countries (Greene et al., 2023). A study of individual PM+ conducted in Colombia near the Venezuelan border found high rates of intervention completion, which the authors partially attributed to the flexibility of conducting sessions at the participant’s home or other locations that accommodated their schedules (Perera et al., 2022), a strategy that is less feasible for a group intervention. To date, most PM+ studies examining implementation and scalability strategies have focused on the individual as opposed to the group format (Spaaij et al., 2023; Woodward et al., 2023). Further adaptations to implementation strategies for group interventions targeting refugee and migrant communities in Colombia and similar contexts where population mobility, insecurity and other factors that present challenges to attendance and retention are needed. Another difference is this study’s focus on women. Although women comprise the majority of study populations from previous gPM+ trials (67–82%) and prior studies have typically delivered gPM+ by gender-matched facilitators in gender-specific groups (Jordans et al., 2021; Acarturk et al., 2022; Bryant et al., 2022a), this is the first study to specifically adapt and test gPM+ among women. Stratified analyses from previous studies have identified possible gender differences favoring the effect of gPM+ among women (Jordans et al., 2021). Therefore, findings from the current study may not be generalizable to men.

This study was designed to build on existing evidence for gPM+ by examining different implementation models. We found that it may be feasible to implement gPM+ using a lower-resource, community-embedded implementation model. While the implications for scalability and sustainability are promising, these findings must be interpreted considering the following limitations. First, the study design and sample size preclude definitive conclusions about the relative cost of these implementation models. The sample size was small and the lagged implementation study design may have introduced bias into our estimates and our results may have been confounded by temporal factors. Relatedly, the study had low rates of intervention completion that differed by study condition. This may have resulted from the long run-in time for participants allocated to the nonspecialized technical support condition, which is supported by the finding that assessment response rates were comparable across study conditions across the study period. This type of design presents challenges in a mobile population, such as the one studied in this research. Further research improving upon this design or testing similar research questions with other designs less prone to these challenges would strengthen the preliminary evidence generated from this study. Despite these limitations, this study has important implications for research and practice.

## Conclusions

This study advances current scientific knowledge on the training and supervision strategies for scalable interventions such as gPM+ to address the mental health needs of people in humanitarian contexts. This study provides preliminary evidence that the quality of implementation of community-based MHPSS interventions delivered through task sharing models may be maintained with fewer specialized resources. These results are relevant for the future implementation of PM+ in its individual and group version with migrants and refugees. They suggest that building the capacity of migrants and refugees through community-centered implementation models has potential to promote the cultural relevance, acceptability and use of MHPSS interventions, and may provide a pathway toward strengthening self- and group-efficacy, social support and wellbeing in mobile populations. The implementation strategies operationalized in this study may serve as a roadmap for delivering scalable interventions delivered by members of the community, especially among people affected by adversities, in contexts where there is limited access to mental health services and psychosocial support, as is the case in Colombia. Further research designed to compare the effectiveness and implementation of diverse community-based MHPSS implementation models on a larger scale and with different population groups is needed.

## Supporting information

Greene et al. supplementary material Greene et al. supplementary material

## Data Availability

The datasets used and/or analyzed during the current study are available from the corresponding author on reasonable request.

## List of abbreviations

ENACT: enhancing assessment of common therapeutic factors
gPM+: Group Problem Management Plus
MHPSS: mental health and psychosocial support
NGO: nongovernmental organization
PTSD: post-traumatic stress disorder

## Supplementary material

The supplementary material for this article can be found at http://doi.org/10.1017/gmh.2024.95.
