# Supplementary material for: Comparing implementation strategies for training and supervising nonspecialists in Group Problem Management Plus: A hybrid effectiveness-implementation trial in Colombia
Source: Glob Ment Health (Camb). 2024 Oct 22;11:e90. doi: 10.1017/gmh.2024.95 (PMC11504925; doi:10.1017/gmh.2024.95)
Supplement: Greene et al. supplementary material [file S2054425124000955sup001.docx]

**Supplemental Table 1. Characteristics of study sample**

|  | **Overall**  **T_0_ (n=128)^a^** | **PM+ with specialized technical support** |  | **PM+ with non-specialized technical support** | |
| --- | --- | --- | --- | --- | --- |
|  |  | **T_0_ (n=68)** |  | **T_0_ (n=59)** | **T_2_ (n=41)** |
| **Age (in years), M (SD)** | 33.26 (10.65) | 31.38 (9.00) |  | 35.29 (12.06) | 34.10 (11.76) |
| **Nationality, n(%)** |  |  |  |  |  |
| Colombian | 10 (8.00) | 4 (6.06) |  | 6 (10.17) | 5 (12.20) |
| Colombian-Venezuelan | 11 (8.00) | 5 (7.58) |  | 6 (10.17) | 5 (12.20) |
| Venezuelan | 104 (83.20) | 57 (86.36) |  | 47 (79.66) | 31 (75.61) |
| **Legal status^b^, n (%)** |  |  |  |  |  |
| *Host community* | 8 (6.30) | 3(4.41) |  | 5(8.47) | 3(7.32) |
| *Internally displaced person* | 4 (3.15) | 2(2.94) |  | 2(3.39) | 0(0.00) |
| *Colombian returnee* | 3 (2.36) | 0(0.00) |  | 3(5.08) | 3(7.32) |
| *Migrant* | 110 (86.61) | 62(91.18) |  | 48(81.36) | 34(82.93) |
| *Asylum seeker* | 1 (0.79) | 0(0.00) |  | 1(1.69) | 0 (0.00) |
| *Refugee* | 1 (0.79) | 1 (1.47) |  | 0 (0.00) | 1(2.44) |
| **Irregular migration status^c^ (among migrants, asylum seekers, refugees), n (%)** | 29 (26.36) | 13 (20.97) |  | 16 (33.33) | 10 (28.57) |
| **Education level, n (%)** |  |  |  |  |  |
| *Less than primary school* | 10 (7.81) | 3 (5.08) |  | 7 (10.29) | 1 (2.44) |
| *Primary school* | 62 (48.44) | 31 (52.54) |  | 31 (45.59) | 20 (48.78) |
| *Bachillerato/High school* | 44 (34.38) | 19 (32.20) |  | 24 (35.29) | 16 (39.02) |
| *Technical school* | 8 (6.25) | 3 (5.08) |  | 5 (7.35) | 2 (4.88) |
| *Pregrado/College or Postgrado/Graduate Degree* | 4 (3.15) | 3 (5.08) |  | 1 (1.47) | 2 (4.88) |
| **Employment, n (%)** |  |  |  |  |  |
| *Unemployed* | 15 (11.90) | 6(8.96) |  | 9(15.25) | 15 (36.59) |
| *Student, volunteer, responsible for the household, or other* | 46 (36.51) | 19 (28.36) |  | 27 (45.76) | 13 (31.70) |
| *Informal work* | 30 (23.81) | 20(29.85) |  | 10(16.95) | 10 (24.39) |
| *Self-employed* | 25 (19.84) | 16(23.88) |  | 9(15.25) | 2 (4.88) |
| *Salaried or formal work* | 10 (7.94) | 6(8.96) |  | 4(6.78) | 1 (2.44) |
| **Marital status, n (%)** |  |  |  |  |  |
| *Never married* | 37 (29.13) | 19(27.94) |  | 18(30.51) | 15 (36.59) |
| *Married or living with partner* | 69 (54.33) | 40 (58.82) |  | 29 (49.15) | 23 (56.00) |
| *Separated, divorced, or widowed* | 21 (16.53) | 9 (13.13) |  | 12 (20.33) | 3 (7.32) |
| **LGBTQI+, n (%)** | 4 (3.17) | 3 (4.48) |  | 1 (1.69) | 0 (0.00) |
| **Household assets, n (%)** |  |  |  |  |  |
| *Electricity* | 124 (97.64) | 65 (95.59) |  | 59 (100.00) | 38 (92.68) |
| *Potable water* | 99 (77.95) | 52 (76.47) |  | 47 (79.66) | 37 (90.24) |
| *Radio* | 54 (42.52) | 28 (41.18) |  | 26 (44.07) | 18 (43.90) |
| *Television* | 91 (72.22) | 51 (75.00) |  | 40 (68.97) | 30 (73.17) |
| *Cell phone* | 113 (90.40) | 62 (91.18) |  | 51 (89.47) | 38 (92.68) |
| *Gas stove* | 54 (42.52) | 25 (36.76) |  | 29 (49.15) | 17 (41.46) |
| *Cement house* | 52 (40.94) | 22 (32.35) |  | 30(50.85) | 14 (34.15) |
| **Prior use of MHPSS services, n (%)** | 25 (19.84) | 14 (20.59) |  | 11 (18.97) | 12 (29.27) |
| **Living with a disability, n (%)** | 7 (5.55) | 3 (4.48) |  | 4 (6.77) | 1 (2.44) |
| **Past-year history of gender-based violence, n (%)** | 24 (19.83) | 15 (22.73) |  | 9 (16.36) | 4 (10.53) |

^a^One participant completed a baseline, but was not randomized. Therefore, their data only contribute to overall sample characteristics

^b^Legal status was ascertained by asking participants how they self-identify and was not based on an official legal determination.

^c^Irregular migration is formally defined as those who are residing in Colombia who are not legally authorized to remain in the country (Bitar, 2022). In the current study, migration status (regular vs. irregular) was self-reported.

**Supplemental Table 2. Change in mental health outcomes over time**

|  | **Specialized training/supervision condition** | |  | | **Non-specialized training/supervision condition** | |  | | **Objective 1. Specialized vs. Non-specialized Group PM+ (Exposure Time)**  *Between-group difference in mean change (95% CI)*^a^ | |  | | **Objective 3. Group PM+ vs. Waitlist (Calendar Time)**  *Between-group difference in mean change (95% CI)* |
| --- | --- | --- | --- | --- | --- | --- | --- | --- | --- | --- | --- | --- | --- |
|  | Mean (SD) | Mean change from T_0_ (95% CI) | | Mean (SD) | | Mean change from T_0_ (95% CI) | | Pre-intervention to endline | | Pre-intervention to 3-month post-intervention | | T_0_ to T_2_ | |
| ***Depressive symptoms (PHQ-9)*** | | | | | | | | UN: -0.29 (-2.96, 2.38)  ADJ: -0.27 (-2.96, 2.42) | | UN: -0.08 (-1.50, 1.35)  ADJ: 0.05 (-1.39, 1.48) | | UN: -0.37 (-1.65, 0.92)  ADJ: -0.42 (-1.71, 0.88) | |
| Baseline, T_0_ | 8.03 (5.26) | REF | | 6.75 (4.49) | | REF | |  | |  | |  | |
| Endline (Specialized), T_1_ | 5.89 (5.03) | -2.21 (-3.97, -0.45)* | | -- | | -- | |  | |  | |  | |
| 3-months post-enrollment, T_2_ | 7.33 (6.10) | -1.02 (-3.14, 1.10) | | 6.51 (5.37) | | -0.46 (-2.32, 1.39) | |  | |  | |  | |
| Endline (Non-specialized), T_3_ | -- | -- | | 4.70 (4.75) | | -2.39 (-4.63, -0.15)* | |  | |  | |  | |
| 6-months post-enrollment, T_4_ | 7.38 (6.96) | -0.22 (-3.20, 2.76) | | 5.79 (4.55) | | -0.90 (-3.12, 1.32) | |  | |  | |  | |
| ***PTSD symptoms (PCL-5)^b^*** | | | | | | | | UN: 0.30 (-0.53, 0.84)  ADJ: 0.28 (-0.26, 0.83) | | UN: -0.14 (-0.45, 0.16)  ADJ: -0.14 (-0.44, 0.17) | | UN: -0.12 (-0.39, 0.16)  ADJ: -0.13 (-0.41, 0.14) | |
| Baseline, T_0_ | 18.60 (16.18) | REF | | 14.92 (14.17) | | REF | |  | |  | |  | |
| Endline (Specialized), T_1_ | 13.30 (12.49) | -5.79 (-10.54, -1.04)* | | -- | | -- | |  | |  | |  | |
| 3-months post-enrollment, T_2_ | 14.76 (17.24) | -5.94 (-11.60, -0.27)* | | 13.12 (13.54) | | -2.66 (-6.75, 1.44) | |  | |  | |  | |
| Endline (Non-specialized), T_3_ | -- | -- | | 9.12 (14.19) | | -6.55 (-11.85, -1.24)* | |  | |  | |  | |
| 6-months post-enrollment, T_4_ | -- | -- | | 11.34 (13.77) | | -4.00 (-10.79, 2.79) | |  | |  | |  | |
| ***Personally identified problems (PSYCHLOPS)*** | | | | | | | | UN: -1.03 (-3.73, 1.67)  ADJ: -0.96 (-3.67, 1.74) | | UN: 0.56 (-0.91, 2.03)  ADJ: 0.59 (-0.88, 2.07) | | UN: 0.12 (-1.15, 1.39)  ADJ: 0.11 (-1.17, 1.39) | |
| Baseline, T_0_ | 13.19 (5.35) | REF | | 13.23 (4.82) | | REF | |  | |  | |  | |
| Endline (Specialized), T_1_ | 11.84 (5.65) | -1.80 (-3.54, -0.05)* | | -- | | -- | |  | |  | |  | |
| 3-months post-enrollment, T_2_ | 12.76 (5.35) | -1.35 (-3.22, 0.53) | | 12.14 (5.21) | | -0.85 (-2.96, 1.26) | |  | |  | |  | |
| Endline (Non-specialized), T_3_ | -- | -- | | 11.74 (4.98) | | -0.51 (-3.01, 2.00) | |  | |  | |  | |
| 6-months post-enrollment, T_4_ | -- | -- | | 10.33 (6.45) | | -1.56 (-4.51, 1.38) | |  | |  | |  | |
| ***Harmful alcohol use (AUDIT) ^b^*** | | | | | | | | UN: -0.14 (-0.45, 0.17)  ADJ: -0.17 (-0.49, 0.14) | | UN: -0.17 (-0.35, 0.00)  ADJ: -0.18 (-0.35, -0.01)* | | UN: -0.10 (-0.25, 0.06)  ADJ: -0.10 (-0.25, 0.06) | |
| Baseline, T_0_ | 2.03 (4.11) | REF | | 1.05 (1.91) | | REF | |  | |  | |  | |
| Endline (Specialized), T_1_ | 1.04 (1.78) | -0.67 (-1.45, 0.12) | | -- | | -- | |  | |  | |  | |
| 3-months post-enrollment, T_2_ | 1.12 (2.84) | -0.67 (-1.63, 0.28) | | 0.78 (1.19) | | -0.24 (-0.93, 0.44) | |  | |  | |  | |
| Endline (Non-specialized), T_3_ | -- | -- | | 1.03 (2.19) | | -0.03 (-0.96, 0.90) | |  | |  | |  | |
| 6-months post-enrollment, T_4_ | -- | -- | | 1.51 (2.63) | | 0.95 (-0.01, 1.91) | |  | |  | |  | |
| ***Migration-related distress (PMLDC)^c^*** | | | | | | | | UN: -3.80 (-9.04, 1.43)  ADJ: -3.85 (-9.04, 1.35) | | UN: -2.60 (-5.32, 0.11)  ADJ: -2.56 (-5.26, 0.15) | | UN: 0.10 (-2.31, 2.51)  ADJ: 0.11 (-2.29, 2.52) | |
| Baseline, T_0_ | 44.83 (11.89) | REF | | 47.88 (11.32) | | REF | |  | |  | |  | |
| Endline (Specialized), T_1_ | 40.42 (10.32) | -3.36 (-6.71, -0.01)* | | -- | | -- | |  | |  | |  | |
| 3-months post-enrollment, T_2_ | 38.40 (10.03) | -4.93 (-8.86, -1.01)* | | 41.30 (10.44) | | -5.14 (-8.79, -1.50)* | |  | |  | |  | |
| Endline (Non-specialized), T_3_ | -- | -- | | 41.34 (10.82) | | -5.62 (-9.07, -2.17)* | |  | |  | |  | |
| 6-months post-enrollment, T_4_ | -- | -- | | 40.20 (9.67) | | -5.53 (-10.46, -0.59)* | |  | |  | |  | |
| ***COVID-19 stress (PSS)*** | | | | | | | | UN: -5.94 (-9.57, -2.31)  ADJ: -6.20 (-9.77, -2.62) | | UN: -5.03 (-6.92, -3.14)  ADJ: -5.07 (-6.96, -3.18) | | UN: 1.40 (-0.27, 3.08)  ADJ: 1.36 (-0.31, 3.02) | |
| Baseline, T_0_ | 10.62 (7.25) | REF | | 11.25 (6.50) | | REF | |  | |  | |  | |
| Endline (Specialized), T_1_ | 7.23 (6.85) | -3.46 (-5.88, -1.03)* | | -- | | -- | |  | |  | |  | |
| 3-months post-enrollment, T_2_ | 7.08 (7.45) | -4.55 (-7.19, -1.91)* | | 4.51 (6.74) | | -6.85 (-9.29, -4.41) | |  | |  | |  | |
| Endline (Non-specialized), T_3_ | -- | -- | | 7.06 (7.45) | | -4.67 (-7.46, -1.88) | |  | |  | |  | |
| 6-months post-enrollment, T_4_ | -- | -- | | 10.59 (6.84) | | -1.07 (-4.33, 2.20) | |  | |  | |  | |

Abbreviations: UN: Unadjusted, ADJ: Adjusted

^a:^Pre-intervention is represented by T_0_ in specialized training/supervision condition and T_3_ in non-specialized training/supervision condition. Endline is represented by T_1_ in the specialized training/supervision condition and T_4_ in non-specialized training/supervision condition. Three-months post-intervention is represented by T_3_ in the specialized training/supervision condition and T_5_ in non-specialized training/supervision condition.

^b^PTSD symptoms and harmful alcohol use were log-transformed in mixed-effects models due to non-normal distributions

^c^Post-migration living difficulties questionnaire only administered to people who identified as a migrant, refugee, asylum seeker, internally displaced person, or returnee

**p*<0.05

**Supplemental Table 3. Adaptations to Group PM+ and its implementation in Barranquilla, Colombia**

| Principle | Subdomain | Description and rationale | When | Type | Source |
| --- | --- | --- | --- | --- | --- |
| Concepts | Addressing stressors and stigma | Expanded eligibility from experience of GBV to all women who were broadly considered at-risk for GBV to reduce stigma and risks related to participation | Pre-implementation | Outer context | Qualitative interviews |
| Context | Increase accessibility | Recommended to ensure representation of key subgroups: LGBTQI+ persons and adolescents. Adolescents were not included due to restrictions related to consent and safety concerns | Pre-implementation | Outer context | Qualitative interviews |
| Context | Ensure compliance | Excluded migrants in transit and those who were likely to leave the city due to risk of attrition and challenges with adherence | Pre-implementation | Outer context | Qualitative interviews |
| Context | Social concepts | Employ a community-based approach that considers the community at the center of the intervention as opposed to a medical model. | Pre-implementation | Process | Qualitative interviews |
| Context | Ensure compliance | Created brief flyers covering main points of each session to ensure continuity and access to information for women who faced barriers to attendance and to promote retention | Implementation | Process | Supervisors and research team observations |
| Goals | Clarifying goals | Assemble groups of 6-8 women to avoid groups becoming to small due to anticipated high rates of attrition | Pre-implementation | Process | Manual review |
| Language | Translation | Align references to an individual’s gender in the manual’s examples to female to correspond to the gender of study participants. For example, change ‘el facilitador’ to ‘la facilitadora’ | Pre-implementation | Intervention characteristics | Manual review |
| Language | Translation; Use of local idioms | Maintain conceptual equivalence of mental health terms and avoid literal or technical translations that distort meaning and/or are stigmatizing, particularly related to mental health terms. For examples “psychological difficulties” was replaced with “problems” | Pre-implementation | Intervention characteristics | Qualitative interviews |
| Language | Translation: Technical terms replaced by colloquialisms | Several terms phrases had to be adapted to fit the context and promote comprehensibility. For example, we adjusted terms describing education levels, cohabitation, taking coffee (instead of tea), | Pre-implementation | Intervention characteristics | Manual review |
| Metaphors | Use of stories and local examples | Adjusted the stories, images, and examples to be reflective of the context, especially those included in the beginning of the intervention | Pre-implementation | Intervention characteristics | Qualitative interviews |
| Methods | Structural adaptation | Intervention sessions took place within secure spaces within community to make the sessions more convenient to attend. Some key informants recommended conducting the sessions outside the community to enable participants to talk more openly and calmly about their problems. | Pre-implementation | Inner setting | Qualitative interviews |
| Methods | Client engagement | Facilitators were trained to understand that the objective of Group PM+ is to benefit diverse communities. As such, participants of diverse backgrounds should be accepted into the groups and respected. Facilitators should maintain a neutral position to avoid cultural, political, or religious disagreements. The facilitator should also serve as a mediator to ensure that all participant’s participate equally in the group sessions. | Pre-implementation | Characteristics of individual | Qualitative interviews; Manual review |
| People | Therapist-patient matching | PM+ facilitators were selected to be women | Pre-implementation | Characteristics of individuals | Qualitative interviews |
| People | Use of non-mental health professionals; Cultural competency of therapists | PM+ facilitators did not need to be mental health professionals, but it was recommended that they have basic knowledge of mental health principles and experience working with women in their community. They could be women with lived experience of gender-based violence or mental health problems who have received support and/or treatment. Community leaders recommended that facilitators have a strong relationship with the community because women would have more confidence with someone they already know | Pre-implementation | Characteristics of individuals | Qualitative interviews |
| People | Use of non-mental health professionals | Created materials for the facilitator to promote visibility motivation, and group identity (e.g., t-shirt, project materials) | Implementation | Characteristics of individuals | Facilitators; Supervisor and research team observations |
| Security | Conflict-affected setting | Participants were contacted by community providers and people they trust about participating in the program. | Pre-implementation | Process | Qualitative interviews |

**Supplemental Table 4. Summary of costs by study condition (in Colombian Pesos, COP)**

| **Cost** | **Cost of implementation** | | |
| --- | --- | --- | --- |
|  | Overall | Specialized training/ supervision condition | Non-specialized training/ supervision condition |
| Total cost of implementation | 280,804,200  *total study cost* | 169,938,000  *cost per arm* | 110,866,200  *cost per arm* |
| *Training costs* | 24,718,000 | 7,916,000 | 16,262,000 |
| Group PM+ Training of Facilitators/Trainers |  | 2,000,000 | 8,462,000 |
| Group PM+ Practice Rounds |  | 5,916,000 | 7,800,000 |
| *Program personnel costs* | 106,424,000 | 88,292,000 | 18,132,000 |
| Supervisor |  | 81,400,000 | 11,240,000 |
| Facilitators |  | 6,892,000 | 6,892,000 |
| *Research personnel costs (research assistants)* | 132,200,000 | 70,000,000 | 62,200,000 |
| Research Assistants | 132,200,000 | 70,000,000 | 62,200,000 |
| *Assessments* | 2,460,000 | 1,230,000 | 1,230,000 |
| Phone credit/telecommunication | 2,460,000 | 1,230,000 | 1,230,000 |
| *Materials and supplies* | 15,542,200 | 2,500,000 | 13,042,200 |
| PM+ Manuals |  | 500,000 | 500,000 |
| Other costs (stationary, materials, supplies, personal protective equipment) |  | 2,000,000 | 12,542,200 |

^a^Exchange rate: 0.00024 USD to 1 COP (2023)

Footnote: Higher personnel costs in the specialized support condition compared to the non-specialized support condition were drive by higher salaries for specialized trainers and supervisors. Other training costs were higher in the non-specialized as compared to the specialized support condition because the new trainers had to secure additional materials, rent a space for the training, and there was an additional training-of-trainers. The cost of materials and supplies was higher in the non-specialized as compared to the specialized support condition because the trainers and facilitators implementing gPM+ within the non-specialized support condition generated new ideas for materials and resources to facilitate implementation, which included facilitator uniforms, take-home materials for Group PM+ participants, and new posters and banners.

**Supplementary Table 5. Description of assessment timepoints and measurement tools**

| **Construct** | **Instrument** |  |  | **Assessment Timepoints** | | | | | | |  |
| --- | --- | --- | --- | --- | --- | --- | --- | --- | --- | --- | --- |
|  |  | **Screening** | **Baseline** | | | **Endline,** *Specialized condition only* | **3m follow-up** | **Endline,**  *Non-specialized condition only* | **6m follow-up** |  |  |
| ***Primary outcome*** | | | | | | | | | |  |  |
| Depression Symptoms | *Patient Health Questionnaire (PHQ-9)* |  | X | | X | | X | X | X |  |  |
| ***Secondary outcome and other measures*** | | | | | | | | | | | |
| General Psychological Distress | *General Health Questionnaire (GHQ-12)* | X | X | | X | | X | X |  |  |  |
| Daily Functioning | *WHODAS* | X | X | | X | | X | X |  |  |  |
| General Psychological Distress | *Reducing Tension Checklist (RTC)* |  | X | | X | | X | X |  |  |  |
| Post-traumatic Stress Symptoms | *PTSD Checklist (PCL-5)* |  |  | | X | | X | X |  |  |  |
| Personalized Outcome | *Psychological Outcome Profiles (PSYCLOPS)* |  | X | | X | | X | X |  |  |  |
| Use of PM+ Skills | *Reducing Tension Checklist (RTC)* |  | X | | X | | X | X |  |  |  |
| Traumatic Events | *Traumatic Events Inventory (TEI)* |  | X | |  | |  |  |  |  |  |
| Gender-based Violence | *ASIST-GBV* |  | X | |  | |  |  |  |  |  |
| Suicidality | *Suicidality* | X | X | |  | |  |  |  |  |  |
| Migration related distress | *Post-Migration Living Difficulties (PMLD)* |  | X | | X | | X | X |  |  |  |
| Stress related to Covid-19 | *Perceived Stress Scale (PSS-10) of Covid-19 Pandemic* |  | X | | X | | X | X |  |  |  |
| Alcohol use disorder | *Alcohol Use Disorders Identification Test (AUDIT)* |  | X | | X | | X | X |  |  |  |
