# Supplementary material for: Comparing implementation strategies for training and supervising nonspecialists in Group Problem Management Plus: A hybrid effectiveness-implementation trial in Colombia
Source: Glob Ment Health (Camb). 2024 Oct 22;11:e90. doi: 10.1017/gmh.2024.95 (PMC11504925; doi:10.1017/gmh.2024.95)
Supplement: Peer review history

# Editor report: Recommendation: Comparing implementation strategies for training and supervising nonspecialists in Group Problem Management Plus: A hybrid effectiveness-implementation trial in Colombia — R0/PR2

**DOI:** 10.1017/gmh.2024.95.pr2

**Affiliations:** Director, National Institute of Mental Health, Czech Republic

No accompanying comment.

# Editor report: Decision: Comparing implementation strategies for training and supervising nonspecialists in Group Problem Management Plus: A hybrid effectiveness-implementation trial in Colombia — R0/PR3

**DOI:** 10.1017/gmh.2024.95.pr3

**Affiliations:** Johns Hopkins University Bloomberg School of Public Health, United States

No accompanying comment.

# Editor report: Recommendation: Comparing implementation strategies for training and supervising nonspecialists in Group Problem Management Plus: A hybrid effectiveness-implementation trial in Colombia — R1/PR5

**DOI:** 10.1017/gmh.2024.95.pr5

**Affiliations:** Director, National Institute of Mental Health, Czech Republic

No accompanying comment.

# Editor report: Decision: Comparing implementation strategies for training and supervising nonspecialists in Group Problem Management Plus: A hybrid effectiveness-implementation trial in Colombia — R1/PR6

**DOI:** 10.1017/gmh.2024.95.pr6

**Affiliations:** Johns Hopkins University Bloomberg School of Public Health, United States

No accompanying comment.
